# Correlation and Survival Analysis of Distant Metastasis Site and Prognosis in Patients With Hepatocellular Carcinoma

**DOI:** 10.3389/fonc.2021.652768

**Published:** 2021-05-10

**Authors:** Hao Zhan, Xue Zhao, Zhaoxue Lu, Yuanhu Yao, Xuguang Zhang

**Affiliations:** ^1^ School of Clinical, Graduate School of Xuzhou Medical University, Xuzhou, China; ^2^ Department of Radiotherapy, Xuzhou Cancer Hospital, Xuzhou, China

**Keywords:** hepatocellular carcinoma, distant metastasis, prognosis, survival analysis, SEER database

## Abstract

**Purpose:**

To investigate the prognostic factors and survival analysis of patients with hepatocellular carcinoma with distant metastasis.

**Methods:**

The clinical data of 3,126 patients with distant metastasis of hepatocellular carcinoma from 2010 to 2015 were extracted from SEER database, and the correlation between the location of distant metastasis of hepatocellular carcinoma and prognosis was retrospectively analyzed. Patients were grouped according to different metastatic sites. The clinical characteristics of each group were compared by chi-square test, the survival curve was drawn by Kaplan-Meier method, Log-rank test was used for univariate analysis, and Cox regression for multivariate analysis. And use propensity score matching (PSM) to reduce differences in baseline characteristics.

**Results:**

Before PSM, the prognosis of patients with hepatocellular carcinoma with lung metastasis is worse than that of patients without lung metastasis. And there was no statistically significant difference with or without bone metastases.Patients with one type of organ metastasis had better prognosis than those with multiple organ metastasis. Among patients with organ metastasis, bone metastasis has a better prognosis than patients with lung metastasis. After PSM, patients with HCC with bone metastases had a worse prognosis than those without bone metastases (*P*<0.05). Univariate analysis showed that the degree of tumor differentiation, T stage, N stage, primary tumor and metastatic surgery, radiotherapy and chemotherapy, tumor size, single organ metastasis, the number of metastatic organs, and the combination of metastatic organs were related to the prognosis of patients with distant metastasis of hepatocellular carcinoma (*P* < 0.05). Multiariate analysis showed that age ≥52 years old, male, low degree of tumor differentiation, N1 stage, no primary surgery, no chemoradiotherapy, tumor size > 6cm, and multi-organ metastasis were independent influencing factors for poor prognosis in patients with metastatic hepatocellular carcinoma.

**Conclusion:**

The lung is the most common site of distant metastasis of hepatocellular carcinoma. Single organ metastasis has better prognosis than multiple organ metastasis. Age ≥52 years old, male, low degree of tumor differentiation, N1 stage, no primary surgery, no chemoradiotherapy, tumor size > 6cm, and multi-organ metastasis were independent influencing factors for poor overall survival and cancer-specific survival prognosis in patients with metastatic hepatocellular carcinoma.

## Introduction

Hepatocellular carcinoma (HCC) is one of the common malignant tumors, which has the characteristics of high morbidity and mortality. In the global cancer report in 2020, the incidence of HCC ranked sixth, with 906,000 new cases and 830,000 deaths each year, ranking second in male tumor mortality (1). The main risk factors for hepatocellular carcinoma include chronic viral hepatitis, alcoholism, non-alcoholic fatty liver disease, exposure to aflatoxin B1, diabetes, and obesity (2, 3). In countries where there is no routine HCC monitoring program, up to 30%-35% of patients have macrovascular invasion and/or extrahepatic spread at the time of initial diagnosis. The common distant metastatic sites are lung, bone, adrenal gland, brain, etc. Accounting for about 47%, 37%, 12%, and 1%, respectively (4–7).

At present, due to the relatively rare data of HCC metastasis, there are few studies to explore the profile of HCC extrahepatic metastasis, and the pattern of extrahepatic metastasis still needs further clarification. In addition, it is not clear whether different metastatic sites will translate into different clinical outcomes. Therefore, we conducted a retrospective study using the Surveillance, Epidemiology and End Results (SEER) database to analyze the correlation between distant metastasis and prognosis in advanced HCC.

## Material and Methods

### Object of Study

The SEER database began to record detailed information about distant metastases in 2010. Therefore, we used adult (≧18 years) patients diagnosed with HCC with distant metastasis (AJCC staging 7th edition M1) from 2010 to 2015 as the research object. Exclude patients with non-primary tumors, unknown metastatic sites and survival data. This study is based on publicly available clinical data from the SEER database and has been approved for use in the SEER database (16864-NOV2019).

### Data Collection

Collect patient-related information from the SEER database: diagnosis time, age, gender, race, marital status, pathological type, tumor differentiation, T stage, N stage, tumor size, tumor primary site, tumor distant metastasis site, tumor primary Information about the surgical status of the site (including local tumor destruction, liver surgery, non-surgical) and the surgical status of the metastatic site, radiotherapy and chemotherapy, and survival status.

### Statistical Analysis

Use Excel software to preliminarily organize the data, and use X-tile software to select the best cutoff values for age and tumor size. The chi-square test was used to compare the clinical characteristics of each group, the Kaplan-Meier method was used to draw the survival curve, the log-rank test was used for univariate analysis, the COX regression was used for multivariate analysis, the corresponding Hazard Ratio (HR) and 95%Confidence interval (CI) were analyzed. PSM is performed by 1:1. *P <*0.05 (two-sided) was considered statistically significant. All statistical analysis in this study was done using SPSS 26.0 software.

## Results

### General Data Analysis

**Figure 1:**
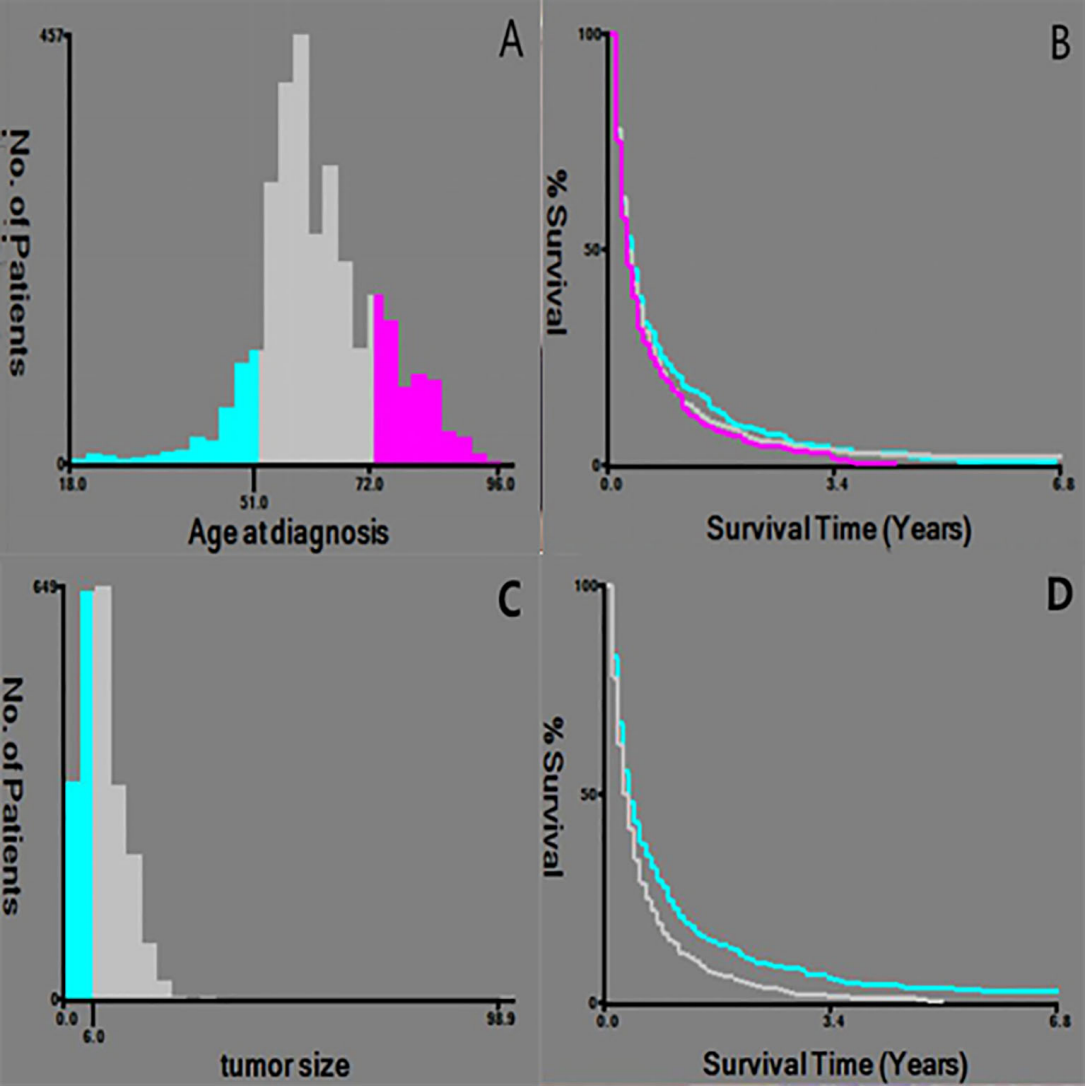
Selection of the best cut-off point for Age and tumor size. **(A, C)** Histogram showing the best cut-off point; **(B, D)** Keplan-Meier curve corresponding to the cut-off point.

A total of 3126 patients with HCC with distant metastasis were included in this study. Among them, there were 1015 cases of bone metastasis (32.47%), 63 cases of brain metastasis (2.02%), and 1175 cases of lung metastasis (37.59%). The age of diagnosis was divided into three groups by X-tile software: ≤51 years old (330 cases, 10.5%), 52-72 years old (2187 cases, 70%), and ≥73 years old (609 cases, 19.5%).Tumor size was divided into three groups: ≤6cm (865 cases, 27.7%), > 6cm (1454, 46.5%), and unknown (807, 25.8%) (**Figure 1**). Non-operative patients with primary tumor and metastatic tumor accounted for the majority, 95.9% and 95.2%, respectively. There were 1740 cases of single organ metastasis (bone, brain, lung, unknown), 240 cases of double organ metastasis (bone + brain, bone + lung, brain + lung), and 11 cases of three organ metastasis. There were only 779 cases of bone metastasis, 18 cases of brain metastasis and 943 cases of lung metastasis.

### Relationship Between Metastatic Sites and Clinical Features

Bone metastasis was correlated with age, race, gender, degree of differentiation, T stage, N stage, metastatic surgery, radiotherapy, and tumor size (*P *< 0.05); Brain metastasis was correlated with differentiation degree, T stage, metastatic surgery, radiotherapy, and chemotherapy (*P *< 0.05); Lung metastasis was correlated with age, race, gender, T stage, N stage, primary tumor surgery and metastatic surgery, radiotherapy, tumor size (*P*<0.05) (**Table 1**).

**Table 1:** The**** relationship between different metastatic sites and clinical features.

Variable	Bone metastasis (n/%)		*P*(χ^2^)	Brain metastasis (n/%)		*P*(χ^2^)	Lung metastasis (n/%)		*P*(χ^2^)
	Yes	No		Yes	No		Yes	No	
	(n=1015)	(n=2111)		(n=63)	(n=3063)		(n=1175)	(n=1951)	
**Age (years)**			0.013			0.229			0.015
18-51	88(8.7)	242(11.5)		8(12.7)	322(10.5)		146(12.4)	184(9.4)	
52-72	743(73.2)	1444(68.4)		48(76.2)	2139(69.8)		792(67.4)	1395(71.5)	
73-96	184(18.1)	425(20.1)		7(11.1)	602(19.7)		237(20.2)	372(19.1)	
**Race**			0.000			0.903			0.000
White	703(69.3)	1398(66.2)		43(68.3)	2058(67.2)		722(61.4)	1379(70.7)	
Black	195(19.2)	321(15.2)		11(17.5)	505(16.5)		211(18.0)	305(15.6)	
Other/unknown	117(11.5)	392(18.6)		9(14.2)	500(16.3)		242(20.6)	267(13.7)	
**Gender**			0.000			0.603			0.002
Male	873(86.0)	1686(79.9)		50(79.4)	2509(81.9)		930(79.1)	1629(83.5)	
Female	142(14.0)	425(20.1)		13(20.6)	554(19.1)		245(20.9)	322(16.5)	
**Marital status**			0.958			0.525			0.727
Married	477(47.0)	1001(47.4)		29(46.0)	1449(47.3)		553(47.1)	925(47.4)	
Unmarried	488(48.1)	1010(47.8)		33(52.4)	1465(47.8)		561(47.7)	937(48,0)	
Other	50(4.9)	100(4.8)		1(1.6)	149(4.9)		61(5.2)	89(4.6)	
**Year of diagnosis**			0.589			0.536			0.565
2010-2012	467(46.0)	993(47.0)		27(42.9)	1433(46.8)		541(46.0)	919(47.1)	
2013-2015	548(54.0)	1118(53.0)		36(57.1)	1630(53.2)		634(54.0)	1032(52.9)	
**Grade stage**			0.000			0.046			0.167
I+II	163(16.1)	439(20.8)		19(30.2)	583(19.0)		217(18.5)	385(19.7)	
III+IV	98(9.7)	295(14.0)		4(6.3)	389(12.7)		164(14.0)	229(11.7)	
Unknown	754(74.2)	1377(65.2)		40(63.5)	2091(68.3)		794(67.5)	1337(68.6)	
**T stage**			0.000			0.001			0.014
T0	10(1.0)	6(0.3)		2(3.2)	14(0.5)		6(0.5)	10(0.5)	
T1	220(21.7)	417(19.8)		20(31.7)	617(20.1)		236(20.1)	401(20.6)	
T2	122(12.0)	230(10.9)		7(11.1)	345(11.3)		124(10.6)	228(11.7)	
T3	374(36.8)	844(40.0)		14(22.2)	1204(39.3)		429(36.5)	789(40.4)	
T4	64(6.3)	272(12.9)		3(4.8)	333(10.8)		153(13.0)	183(9.4)	
TX	225(22.2)	342(16.2)		17(27.0)	550(18.0)		227(19.3)	340(17.4)	
**N stage**			0.000			0.176			0.000
N0	675(66.5)	1260(59.7)		46(73.0)	1889(61.7)		755(64.3)	1180(60.5)	
N1	183(18.0)	571(27.0)		10(15.9)	744(24.3)		230(19.5)	524(26.9)	
Nx	157(15.5)	280(13.3)		7(11.1)	430(14.0)		190(16.2)	247(12.6)	
**Primary tumor surgery**			0.149			0.616			0.001
No	980(96.5)	2006(95.0)		60(95.2)	2926(95.5)		1143(97.3)	1843(94.5)	
Local tumor destruction	16(1.6)	45(2.1)		2(3.2)	59(1.9)		13(1.1)	48(2.5)	
Surgery	19(1.9)	60(2.9)		1(1.6)	78(2.6)		19(1.6)	60(3.0)	
**Metastatic Surgery**			0.000			0.001			0.000
Yes	79(7.8)	71(3.4)		9(14.3)	141(4.6)		33(2.8)	117(6.0)	
No	936(92.2)	2040(96.6)		54(85.7)	2922(95.4)		1142(97.2)	1834(94.0)	
**Radiotherapy**			0.000			0.000			0.000
Yes	495(48.8)	168(8.0)		28(44.4)	635(20.7)		135(11.5)	528(27.1)	
No	520(51.2)	1943(92.0)		35(55.6)	2428(79.3)		1040(88.5)	1423(72.9)	
**Chemotherapy**			0.211			0.018			0.930
Yes	471(46.4)	1030(48.8)		21(33.3)	1480(48.3)		563(47.9)	938(48.1)	
No	544(53.6)	1081(51.2)		42(66.7)	1583(51.7)		612(52.1)	1013(51.9)	
**Tumor size**			0.000			0.176			0.000
≤6	320(31.5)	545(25.8)		21(33.3)	844(27.6)		254(21.6)	611(31.3)	
>6	410(40.4)	1044(49.5)		22(34.9)	1432(46.7)		600(51.1)	854(43.8)	
Unknown	285(28.1)	522(24.7)		20(31.7)	787(25.7)		321(27.3)	486(24.9)	

### Survival Analysis

**Figure 2:**
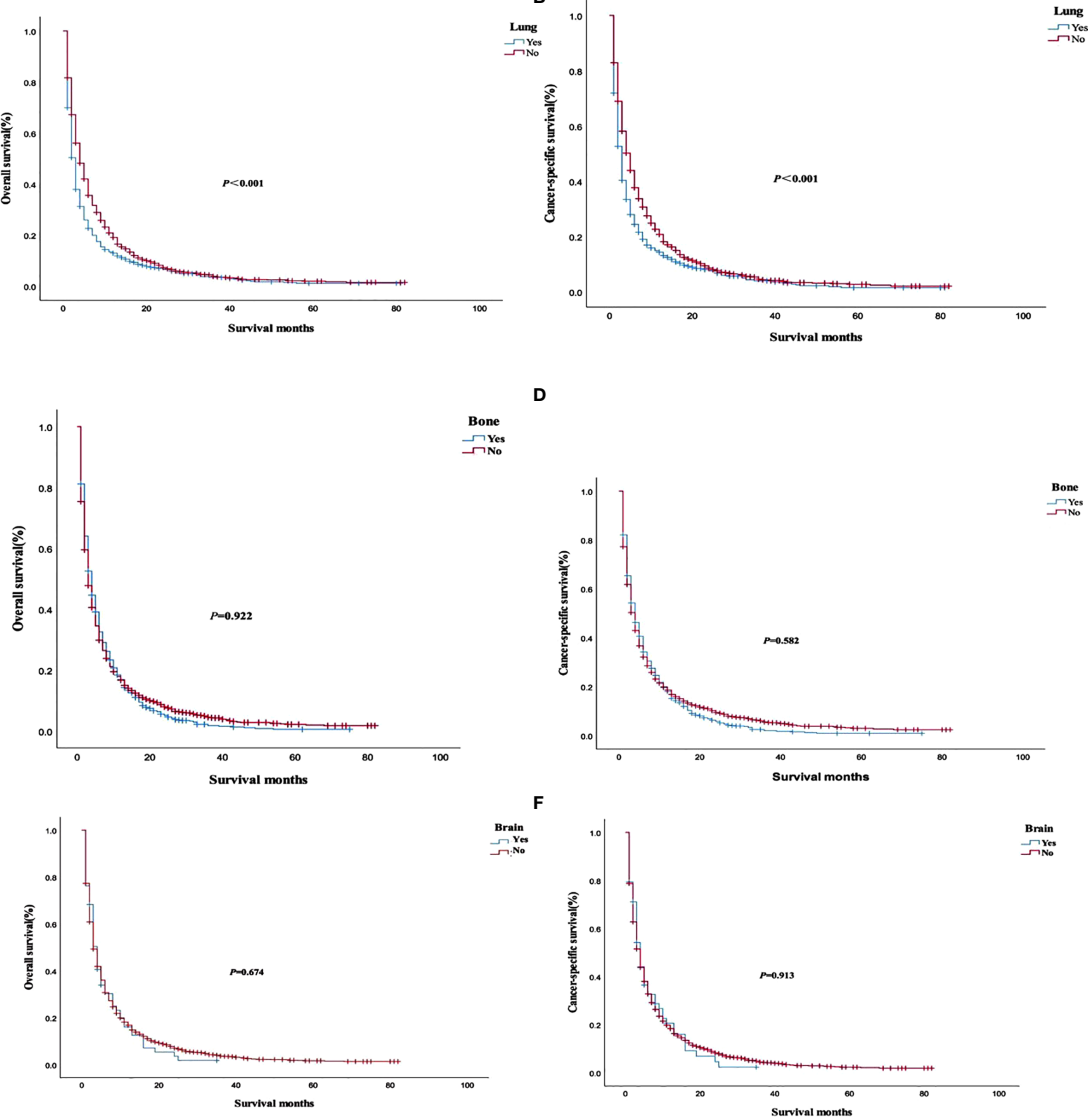
Overall survival and cancer-specific survival curves with or without corresponding organ metastasis. **(A, B)** lung metastasis; **(C, D)** bone metastasis; **(E, F)** brain metastasis.

**Figure 3:**
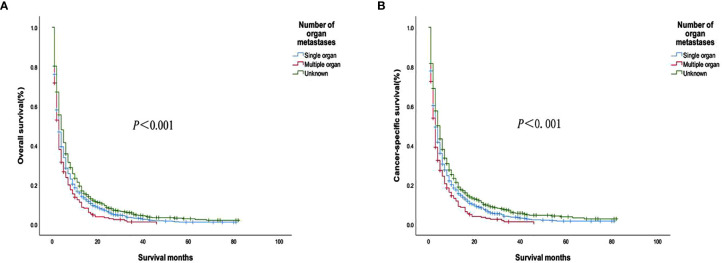
**(A)** Overall survival and **(B)** Cancer-specific survival curves of patients with different metastatic organ numbers.

**Figure 4:**
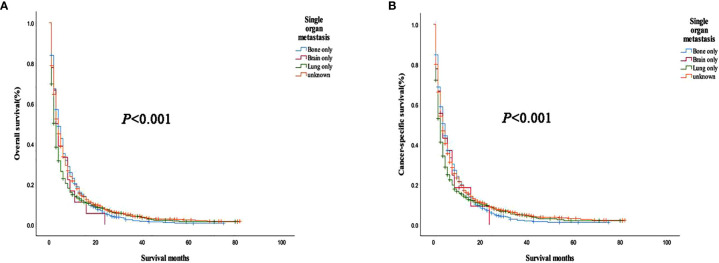
**(A)** Overall survival and **(B)** cancer-specific survival curves of different metastatic sites in a patient with single organ metastasis.

The analysis of the prognosis of distant metastasis showed that OS (*P*<0.001) and CSS (*P*<0.001) were worse than those without lung metastasis. However, there was no significant difference in OS (*P*=0.922; *P* = 0.674) and CSS (*P* = 0.582; *P* = 0.913) in patients with bone and brain metastases than those without bone and brain metastases (**Figure 2**). Patients with more single metastasis had better OS (*P *= 0.003) and CSS (*P *= 0.001) (**Figure 3**). Among single metastases, bone metastases were better than those with lung metastases in OS (*P <*0.001) and CSS (*P <*0.001), but bone metastases and brain metastases, brain metastases and lung metastases OS (*P *= 0.386, *P *= 0.808) and CSS (*P *= 0.620, *P *= 0.624) had no significant difference (**Figure 4**). The 1-year and 3-year survival rates of bone, brain and lung metastases were 18.7%, 5.6%, 13.6% and 1.9%, 0%, 4.9%, respectively. The 1-year and 3-year cancer-specific survival rates were 19.9%, 9.3%, 15.3%, and 2.2%, 0%, 4.9%, respectively.

**Figure 5:**
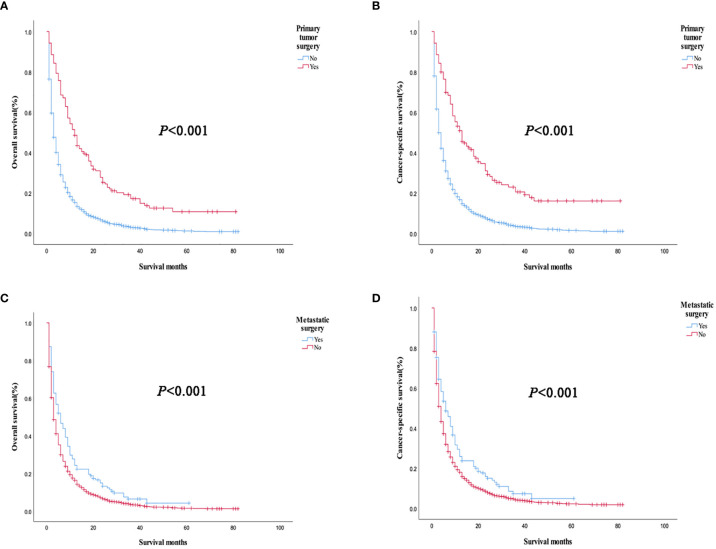
Overall survival and cancer-specific survival curves of primary tumors and metastatic tumors in all patients with organ metastasis. **(A, B)** Primary tumor surgery; **(C, D)** Metastatic surgery.

Prognostic analysis of primary tumor and metastatic surgery showed that OS (*P *< 0.001) and CSS (*P *< 0.001) were improved in patients who underwent primary tumor surgery compared with those who did not. Patients who underwent metastatic surgery had improved OS (*P *< 0.001) and CSS (*P *< 0.001) compared with those who did not (**Figure 5**). Further stratified analysis of primary surgery showed that OS (*P *= 0.006, *P*<0.001) and CSS (*P *= 0.005, *P*<0.001) were improved in patients with bone or lung single-organ metastasis who underwent primary tumor surgery, but there was no significant survival benefit in single-organ brain metastasis (OS: *P *= 0.982, CSS: *P *= 0.904). The results of the analysis of the surgical methods of the primary tumor showed that local tumor destruction had no significant effect on the OS (*P *= 0.902, *P *= 0.648) and CSS (*P *= 0.720, *P*=0.496) of bone and lung metastases compared with liver surgery. Further stratified analysis of metastatic surgery showed that patients with lung metastases had better OS (*P *= 0.023) and CSS (*P *= 0.015), but no significant effect on survival of patients with bone or brain metastases (bone metastases: OS *P *= 0.15, CSS *P *= 0.269; Brain metastases: OS *P* = 0.841, CSS *P* = 0.752).

### Analysis of Influencing Factors

Univariate analysis showed that the degree of tumor differentiation, T stage, N stage, primary tumor and metastatic surgery, radiotherapy and chemotherapy, tumor size, single organ metastasis, the number of metastatic organs, and the combination of metastatic organs were the risk factors that affecting the prognosis of patients with distant metastasis of HCC. According to multivariate analysis, age ≥52 years old, male, low differentiation, N1 stage, no primary surgical resection, no chemoradiotherapy, tumor size > 6cm, and multiple organ metastasis were the independent influencing factors for poor prognosis in patients with metastatic HCC (**Table 2**).

**Table 2:** Multivariate analysis of prognosis for distant metastasis of HCC.

Variable	Overall Survival		Cancer-specific Survival	
	HR(95%CI)	*P*	HR(95%CI)	*P*
**Age(years)**				
18-51	1.000(*Reference*)		1.000(*Reference*)	
52-72	1.132(1.003-1.277)	0.045	1.150(1.015-1.303)	0.028
73-96	1.199(1.043-1.380)	0.011	1.212(1.049-1.400)	0.009
**Gender**				
Male	1.000*(Reference)*		1.000*(Reference)*	
Female	0.881(0.800-0.971)	0.011	0.873(0.790-0.966)	0.008
**Grade stage**				
I+II	1.000*(Reference)*		1.000*(Reference)*	
III+IV	1.503(1.315-1.717)	0.000	1.510(1.318-1.729)	0.000
Unknown	1.120(1.018-1.232)	0.020	1.103(1.000-1.216)	0.050
**T stage**				
T0	1.000*(Reference)*		1.000*(Reference)*	
T1	0.752(0.440-1.287)	0.299	0.785(0.450-1.371)	0.395
T2	0.897(0.523-1.538)	0.692	0.918(0.525-1.605)	0.763
T3	0.960(0.561-1.641)	0.881	1.011(0.579-1.763)	0.970
T4	0.976(0.566-1.684)	0.931	1.037(0.589-1.825)	0.901
TX	0.756(0.438-1.307)	0.317	0.804(0.456-1.417)	0.450
**N stage**				
N0	1.000*(Reference)*		1.000*(Reference)*	
N1	1.130(1.032-1.236)	0.008	1.130(1.030-1.240)	0.009
Nx	0.952(0.849-1.067)	0.394	0.937(0.833-1.054)	0.277
**Primary tumor surgery**				
No	1.000*(Reference)*		1.000*(Reference)*	
Local tumor destruction	0.518(0.394-0.682)	0.000	0.487(0.364-0.651)	0.000
Surgery	0.421(0.323-0.548)	0.000	0.386(0.295-0.505)	0.000
**Metastatic surgery**				
Yes	1.000*(Reference)*		1.000*(Reference)*	
No	1.206(1.009-1.441)	0.039	/	0.053
**Radiotherapy**				
Yes	1.000*(Reference)*		1.000*(Reference)*	
No	1.325(1.207-1.455)	0.000	1.318(1.199-1.449)	0.000
**Chemotherapy**				
Yes	1.000*(Reference)*		1.000*(Reference)*	
No	1.660(1.540-1.790)	0.000	1.607(1.488-1.736)	0.000
**Tumor size**				
≤6	1.000*(Reference)*		1.000*(Reference)*	
>6	1.261(1.141-1.394)	0.000	1.256(1.133-1.393)	0.000
Unknown	1.365(1.204-1.547)	0.000	1.365(1.200-1.553)	0.000
**Metastatic site**				
Bone only	1.000*(Reference)*		1.000*(Reference)*	
Brain only	/	0.456	/	0.498
Lung only	/	0.701	/	0.574
Unknown	/	0.218	/	0.271
**Number of metastases**				
Single	1.000*(Reference)*		1.000*(Reference)*	
Multiple	1.221(1.066-1.399)	0.004	1.253(1.091-1.438)	0.001
Unknown	0.811(0.747-0.880)	0.000	0.805(0.740-0.876)	0.000
**Multiple organ metastasis**				
Bone+brain	1.000*(Reference)*		1.000*(Reference)*	
Bone+lung	/	0.573	/	0.350
Brain+lung	/	0.469	/	0.254
Bone+brain+lung	/	0.829	/	0.834
Unknown	/	0.733	/	0.879

### Survival Analysis After PSM

**Figure 6:**
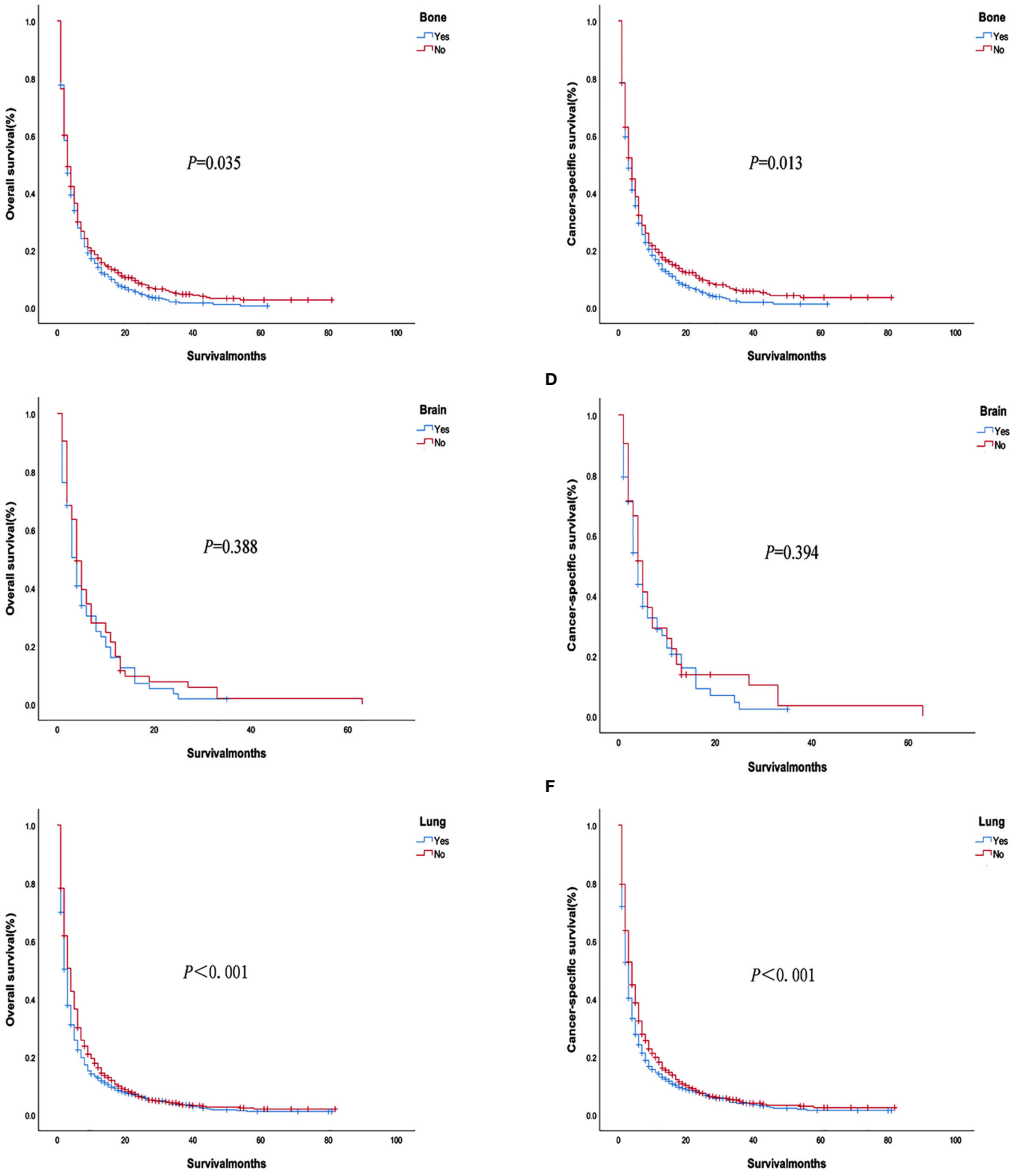
Overall survival and cancer-specific survival curves with or without corresponding organ metastasis after PSM. **(A, B)** Bone metastasis; **(C, D)** Brain metastasis; **(E, F)** Lung metastasis.

**Figure 7:**
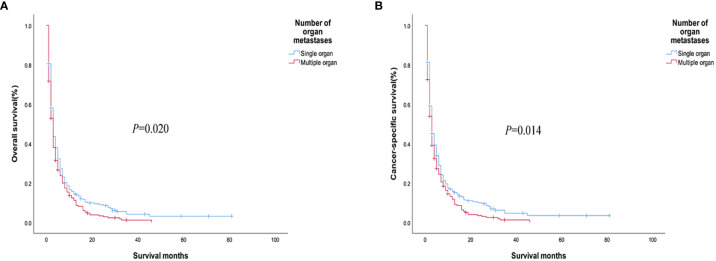
**(A)** Overall survival and **(B)** cancer-specific survival curves of patients with different numbers of metastatic organs after PSM.

We used PSM to analyze the bone, brain, and lung metastasis groups in a 1:1 paired analysis. Among them, there were 687 patients with or without bone metastasis, 63 with or without brain metastasis, and 1169 with or without lung metastasis (**Table 3**). The Kaplan–Meier method was used to draw the survival curve. The results showed that the OS (*P <*0.001; *P*=0.035) and CSS (*P <*0.001, *P*=0.013) were worse in patients with lung, bone metastasis than without lung, and bone metastasis. However, there was no significant difference in OS (*P* = 0.388) and CSS (*P* = 0.394) in patients with brain metastases compared with patients without brain metastases (**Figure 6**); The OS (*P* = 0.020) and CSS (*P* = 0.014) of patients with single metastasis were better than those with multiple metastases (**Figure 7**). The 1-year and 3-year survival rates for bone, brain, and lung metastases were 14.0%, 16.0%, 12.6%, and 1.6%, 0%, and 3.3%, respectively; the 1-year and 3-year cancer-specific survival rates were 15.2%, 16.0%, 14.0% and 1.8%, 0%, 3.9%, respectively. For primary tumor surgery, further stratified analysis showed that OS (*P *= 0.000, *P *= 0.000) and CSS (*P *= 0.000, *P*=0.000) of patients with bone or lung metastases who underwent primary tumor surgery increased. There was no obvious survival benefit for brain metastasis (OS: *P* = 0.104, CSS: *P* = 0.043). The results of the analysis of the surgical methods of the primary tumor showed that the OS (*P *= 0.298, *P *= 0.139, *P *= 0.789) and CSS (*P *= 0.536, *P *= 0.377, *P *= 0.879) of local tumor destruction and liver surgery on bone, brain, and lung metastases had no significant effect. The results of further stratified analysis of metastasis surgery showed that patients with bone and lung metastases had better OS (*P* = 0.003, *P* = 0.001) and CSS (*P* = 0.011, *P* = 0.001). However, there was no significant effect on the survival of patients with brain metastases (OS: *P* = 0.665; CSS: *P* = 0.884).

**Table 3:** The relationship between different metastatic sites and clinical features (after PSM).

Variable	Bone metastasis(n/%)		*P*(χ^2^)	Brain metastasis (n/%)		*P*(χ^2^)	Lung metastasis(n/%)		*P*(χ^2^)
	Yes(n=687)	No(n=687)		Yes(n=63)	No(n=63)		Yes(n=1169)	No(n=1169)	
**Age(years)**			0.604			0.235			0.174
18-51	60(8.7)	65(9.5)		8(12.7)	15(23.8)		145(12.4)	116(9.9)	
52-72	506(73.7)	492(71.6)		48(76.2)	35(55.6)		788(67.4)	836(71.5)	
73-96	121(77.6)	130(18.9)		7(11.1)	13(20.6)		236(20.2)	217(18.6)	
**Race**			0.002			0.190			0.631
White	445(64.8)	472(68.7)		43(68.3)	49(77.8)		722(61.8)	765(65.4)	
Black	148(21.5)	109(15.9)		11(17.5)	5(8.0)		210(18.0)	200(17.1)	
Other/unknown	94(13.7)	106(15.4)		9(14.2)	9(14.2)		237(20.3)	204(17.5)	
**Gender**			0.603			0.348			0.173
Male	582(84.7)	564(82.1)		50(79.4)	53(84.1)		930(80.0)	943(80.7)	
Female	105(15.3)	123(17.9)		13(20.6)	10(15.9)		239(20.0)	226(19.3)	
**Marital status**			0.751			0.153			0.535
Married	304(44.3)	327(47.6)		29(46.0)	27(42.9)		549(47.0)	536(45.9)	
Unmarried	348(50.7)	326(47.5)		33(52.4)	29(46.0)		55947.8()	578(49.4)	
Other	35(5.0)	34(4.9)		1(1.6)	7(11.1)		61(5.2)	55(4.7)	
**Year of diagnosis**			0.516			0.269			0.868
2010-2012	324(47.2)	333(48.5)		27(42,9)	28(44.4)		540(46.2)	530(45.3)	
2013-2015	363(52.8)	354(51.5)		36(57.1)	35(55.6)		629(53.8)	639(54.7)	
**Grade stage**			0.940			0.022			0.382
I+II	129(18.8)	118(17.2)		19(30.2)	12(19.0)		214(18.3)	240(20.5)	
III+IV	77(11.2)	105(15.3)		4(6.3)	4(6.3)		163(13.9)	137(11.7)	
Unknown	481(70.0)	464(67.5)		40(63.5)	47(74.7)		792(67.8)	792(67.8)	
**T stage**			0.000			0.011			0.518
T0	7(1.0)	3(0.4)		2(3.1)	0(0)		6(0.5)	3(0.3)	
T1	138(20.1)	115(16.7)		20(31.7)	14(22.2)		233(19.9)	208(17.8)	
T2	84(12.2)	74(10.8)		7(11.1)	7(11.1)		124(10.6)	125(10.7)	
T3	260(37.8)	283(41.2)		14(22.2)	29(46.0)		428(36.6)	481(41.1)	
T4	42(6.1)	86(12.5)		3(4.8)	5(8.0)		152(13.0)	126(10.8)	
TX	156(22.8)	126(18.3)		17(27.1)	8(12.7)		226(19.3)	226(19.3)	
**N Stage**			0.022			0.730			0.000
N0	450(65.6)	424(61.7)		46(73.0)	35(55.6)		749(64.1)	704(60.2)	
N1	132(19.2)	163(23.7)		10(15.9)	13(20.6)		230(19.7)	318(27.2)	
Nx	105(15.2)	100(14.6)		7(11.1)	15(23.8)		190(16.3)	147(12.6)	
**Primary tumor surgery**			0.600			1.000			0.763
No	663(96.5)	655(95.3)		60(95.2)	60(95.2)		1137(97.3)	1138(97.3)	
Local tumor destruction	9(1.3)	12(1.7)		2(3.2)	3(4.8)		13(1.1)	10(0.9)	
Surgery	15(2.2)	20(3.0)		1(1.6)	0(0)		19(1.6)	21(1.8)	
**Metastatic Surgery**			0.303			0.593			0.714
Yes	43(6.3)	42(6.1)		9(14.3)	5(8.0)		33(2.8)	39(3.3)	
No	644(93.7)	645(93.9)		54(85.7)	5892.0()		1136(97.2)	1130(96.7)	
**Radiotherapy**			0.950			0.064			0.948
Yes	168(24.5)	166(24.2)		28(44.4)	20(31.7)		135(11.5)	137(11.7)	
No	519(75.5)	521(75.8)		35(55.6)	43(68.3)		1034(88.5)	1032(88.3)	
**Chemotherapy**			0.175			0.271			0.709
Yes	302(44.0)	308(44.8)		21(33.3)	26(41.2)		560(47.9)	525(44.9)	
No	385(56.0)	379(55.2)		42(66.7)	37(58.8)		609(52.1)	644(55.1)	
**Tumor size**			0.002			0.444			0.012
≤6	216(31.4)	191(27.8)		21(33.3)	19(30.2)		254(21.7)	306(26.2)	
>6	275(40.0)	308(44.8)		22(34.9)	31(49.2)		598(51.2)	532(45.5)	
Unknown	196(28.6)	188(27.4)		20(31.8)	13(20.6)		317(27.1)	331(28.3)	

## Discussion

HCC is a common malignant tumor of the digestive tract. Since the early symptoms of HCC are not obvious, most patients have already had distant metastasis when diagnosed, thus losing the best chance of treatment and having a poor prognosis. In recent years, most articles have studied the impact of HCC with single-organ distant metastasis (such as bone, brain, lung metastasis) on the prognosis, and most studies are based on a single institution with a small sample size, which greatly limits the implementation of stratified analysis. However, there are few studies on the relationship between different metastasis sites and prognosis. We expanded the cases of patients with distant metastases from 2014 to 2015 on the basis of previous studies based on the SEER database from 2010 to 2013 (8), and analyzed brain metastases on the basis of bone and lung metastases, and further study the impact of tumor size, pathological grade, N stage, radiotherapy and chemotherapy, metastatic organ combination and other related information on the prognosis of patients. Therefore, we used the clinical data of a large number of HCC patients with distant metastases in the SEER database to conduct survival analysis, and explore the clinical characteristics of different metastasis sites and their impact on the prognosis of patients.

The results of this study show that the proportion of distant lung metastases is the highest, followed by bone and brain, which is consistent with the results of previous studies (4, 5). This study believes that compared with older patients, younger patients had a relatively better survival period, and age was an independent factor affecting the prognosis of patients with distant metastases of HCC. Related studies have also shown that age is an important factor affecting the incidence and mortality of HCC. Men over 45 years of age and women over 55 years of age have become a contributing factor for the morbidity and mortality of HCC (9). In this study, male patients with distant HCC metastasis accounted for a large proportion, and multivariate analysis showed that male patients had a worse prognosis than female patients. A retrospective study based on the SEER database from 1973 to 2010 showed that the median OS in women with HCC patients was higher than that in men (10), which was consistent with the conclusion of our study. In this study, the prognosis of single organ metastasis is better, which is consistent with previous research results in lung cancer (11).

The results of this study showed that patients with lower degree of differentiation had worse prognosis, and the mean survival time of patients with lower and undifferentiated differentiation was significantly longer (10.169 months vs.5.862 months, *P *= 0.000), which was consistent with the results of KerCG et al. (12). In addition, our study found that patients with tumors larger than 6 cm were more likely to develop bone and lung metastases, and the prognosis was worse. The incidence of distant metastasis in the group with tumor size ≥58mm was 5.7 times higher than that in the group with tumor size ≤30mm and 2.9 times higher than that in the group with tumor size 30-58mm, which was consistent with the results of this study (13). Therefore, for HCC patients with large tumors, routine chest CT and skeletal ECT examinations are necessary to help early detection of distant metastases.

At present, the value of surgical treatment has been proven in a variety of metastatic solid tumors [such as metastatic non-functional neuroendocrine tumors (14) and metastatic renal cell carcinoma (15)]. Therefore, we analyzed the effect of treatment modality on patient survival, and the results showed that patients with metastatic HCC who did not receive primary surgery and did not receive chemoradiotherapy had a poor prognosis. We further analyzed the impact of primary tumor surgery on the prognosis, and the results showed that there was no significant difference between local tumor destruction and liver surgery on patient survival. For HCC patients with bone metastases, patients who underwent primary surgery had better OS and CSS. Some studies also believed that long-term survival may be related to hepatectomy and radiotherapy of bone metastasis (16); For HCC patients with brain metastasis, the mean survival time was lower than bone and lung metastasis, and the prognosis was poor. At present, a variety of treatment methods including surgery, whole brain radiation therapy (WBRT), and stereotactic radiotherapy (gamma knife, cyberknife, etc.) have been studied to improve survival (17–19); For patients with HCC with lung metastasis, our study found that the OS and CSS of patients receiving primary and metastatic surgery were higher than those without surgery. At present, there are many case reports and clinical studies of radical hepatectomy and lung metastasis for patients with HCC with lung metastases, which are consistent with our findings (20–22). Therefore, for patients diagnosed with HCC, it is recommended to review regularly after surgery to find lung metastases in time. For those who can be surgically removed, surgical treatment is recommended. For patients with metastatic HCC, monotherapy has a poor prognosis, while comprehensive treatment can often achieve better survival. However, further randomized controlled clinical trials are needed to explore the best treatment mode, so as to better guide clinical practice and bring good news to patients.

This study initially explored the relationship between the location of HCC distant metastasis and prognosis, prognostic-related risk factors and survival analysis, but there were still certain limitations. First of all, this study was a retrospective study, and potential bias was inevitable; the data came from the SEER database and was only for the cancer population in the United States. Although the number of cases was large and the follow-up time was relatively complete. However, due to differences in race, region, diet, and environment, the applicability of the results cannot be judged. Therefore, we look forward to further research on large samples of data from various countries to prove it. Secondly, this study only included distant metastases of bone, brain and lung, and did not provide metastases at other sites (such as adrenal gland and peritoneum), so it was impossible to evaluate the impact of metastases in other sites on survival. Finally, due to the lack of systematic treatment details in the SEER database, it is impossible to assess the impact on patient survival.

## Conclusion

In conclusion, the lung is the most common site of distant metastasis of hepatocellular carcinoma. The prognosis of patients with HCC with distant metastasis is poor, and different metastatic sites have different effects on the survival of patients with metastatic HCC. In single-organ metastasis, lung metastasis has a worse prognosis than bone metastasis, and the average survival time of brain metastasis is shorter than bone or lung metastasis. The prognosis of single organ metastasis is good. Age ≥52 years old, male, low degree of differentiation, N1 stage, no primary tumor surgery, no radiotherapy and chemotherapy, tumor size>6cm, and multiple organ metastasis are independent factors that affect the poor prognosis of patients with metastatic HCC. Therefore, for HCC patients with high-risk factors, clinical attention should be paid to early detection, early diagnosis and early treatment to improve the prognosis of patients. In addition, the analysis of the impact of different metastasis sites on the prognosis may provide more evidence for the precise medication and individualized treatment of patients with advanced hepatocellular carcinoma.

## Data Availability Statement

The original contributions presented in the study are included in the article/supplementary material. Further inquiries can be directed to the corresponding author.

## Author Contributions

HZ and XueZ contributed to data acquisition and statistical analysis and prepared the manuscript. ZL helped with data collection. XugZ supervised the study. YY helped a lot in the revision of the article. All authors contributed to the article and approved the submitted version.

## Funding

This study was sponsored by the Natural Science Foundation of Jiangsu Province (BK20151156).

## Conflict of Interest

The authors declare that the research was conducted in the absence of any commercial or financial relationships that could be construed as a potential conflict of interest.

## References

[1] Sung H Ferlay J Siegel RL Laversanne M Soerjomataram I Jemal A. Global Cancer Statistics 2020: GLOBOCAN Estimates of Incidence and Mortality Worldwide for 36 Cancers in 185 Countries. CA Cancer J Clin (2021) 0:1–41. 10.3322/caac.21660 33538338

[2] Perz JF Armstrong GL Farrington LA Hutin YJ Bell BP. The Contributions of Hepatitis B Virus and Hepatitis C Virus Infections to Cirrhosis and Primary Live R Cancer Worldwide. J Hepatol (2006) 45(4):529–38. 10.1016/j.jhep.2006.05.013 16879891

[3] Welzel TM Graubard BI Quraishi S Zeuzem S Davila JA El-Serag HB. Population-Attributable Fractions of Risk Factors for Hepatocellular Carcinoma in the United States. Am J Gastroenterol (2013) 108(8):1314–21. 10.1038/ajg.2013.160 PMC4105976 23752878

[4] Katyal S Oliver JH Peterson MS Ferris JV Carr BS Baron RL. Extrahepatic Metastases of Hepatocellular Carcinoma. Radiology (2000) 216(3):698–703. 10.1148/radiology.216.3.r00se24698 10966697

[5] Uka K Aikata H Takaki S Shirakawa H Jeong SC Yamashina K. Clinical Features and Prognosis of Patients With Extrahepatic Metastases From Hepatocellular Carcinoma. World J Gastroenterol (2007) 13(3):414–20. 10.3748/wjg.v13.i3.414 PMC4065897 17230611

[6] Villanueva A Hernandez-Gea V Llovet JM. Medical Therapies for Hepatocellular Carcinoma: A Critical View of the Evidence. Nat Rev Gastroenterol Hepatol (2013) 10(1):34–42. 10.1038/nrgastro.2012.199 23147664

[7] Mittal S Kanwal F Ying J Chung R Sada YH Temple S. Effectiveness of Surveillance for Hepatocellular Carcinoma in Clinical Practice: A United States Coho Rt. J Hepatol (2016) 65(6):1148–54. 10.1016/j.jhep.2016.07.025 PMC5322857 27476765

[8] Oweira H Petrausch U Helbling D Schmidt J Mehrabi A Schob O. Prognostic Value of Site-Specific Extra-Hepatic Disease in Hepatocellular Carcinoma: A SEER Database Analysis. Expert Rev Gastroenterol Hepatol (2017) 11(7):695–701. 10.1080/17474124.2017.1294485 28276812

[9] Wang F Mubarik S Zhang Y Wang L Wang Y Yu C. Long-Term Trends of Liver Cancer Incidence and Mortality in China 1990-2017: A Joinpoint and Age-Period-Cohort Analysis. Int J Environ Res Public Health (2019) 16(16):2878. 10.3390/ijerph16162878 PMC6719938 31408961

[10] Yang D Hanna DL Usher J LoCoco J Chaudhari P Lenz HJ. Impact of Sex on the Survival of Patients With Hepatocellular Carcinoma: A Surveillance, Epidemiology, and End Results Analysis. Cancer (2014) 120(23):3707–16. 10.1002/cncr.28912 PMC9191612 25081299

[11] Riihimäki M Hemminki A Fallah M Thomsen H Sundquist K Sundquist J. Metastatic Sites and Survival in Lung Cancer. Lung Cancer (2014) 86(1):78–84. 10.1016/j.lungcan.2014.07.020 25130083

[12] Ker CG Chen HY Chen KS Jeng IJ Yang MY Juan CC. Clinical Significance of Cell Differentiation in Hepatocellular Carcinoma. Hepatogastroenterology (2003) 50(50):475–9. 12749251

[13] Yan B Bai DS Zhang C Qian JJ Jin SJ Jiang GQ. Characteristics and Risk Differences of Different Tumor Sizes on Distant Metastases of Hepatocellular Carcinoma: A Retrospective Cohort Study in the SEER Database. Int J Surg (2020) 80:94–100. 10.1016/j.ijsu.2020.06.018 32619622

[14] Keutgen XM Nilubol N Glanville J Sadowski SM Liewehr DJ Venzon DJ. Resection of Primary Tumor Site is Associated With Prolonged Survival in Metastatic Nonfunctioning Pancreatic Neuroendocrine Tumors. Surgery (2016) 159(1):311–8. 10.1016/j.surg.2015.05.042 PMC4688058 26453135

[15] Heng DY Wells JC Rini BI Beuselinck B Lee JL Knox JJ. Cytoreductive Nephrectomy in Patients With Synchronous Metastases From Renal Cell Carcinoma: Results From the International Metastatic Renal Cell Carcinoma Database Consortium. Eur Urol (2014) 66(4):704–10. 10.1016/j.eururo.2014.05.034 24931622

[16] Chao C Imada T Takehana T Morinaga S Miyazaki T Maehara T. [a Case of Hepatocellular Carcinoma With Bone Metastasis Responding to Oral Administration of UFT]. Gan To Kagaku Ryoho (1995) 22(3):403–6. 7880113

[17] Han MS Moon KS Lee KH Cho SB Lim SH Jang WY. Brain Metastasis From Hepatocellular Carcinoma: The Role of Surgery as a Prognostic Factor. BMC Cancer (2013) 13:567. 10.1186/1471-2407-13-567 24289477 PMC3879022

[18] Xu Q Wu P Feng Y Ye K Tong Y Zhou Y. Gamma Knife Surgery for Brain Metastasis From Hepatocellular Carcinoma. PloS One (2014) 9(2):e88317. 10.1371/journal.pone.0088317 24516635 PMC3917852

[19] Que J Kuo HT Lin LC Lin KL Lin CH Lin YW. Clinical Outcomes and Prognostic Factors of Cyberknife Stereotactic Body Radiation Therapy for Unresectable Hepatocellular Carcinoma. BMC Cancer (2016) 16:451. 10.1186/s12885-016-2512-x 27405814 PMC4941022

[20] Mizuguchi S Nishiyama N Izumi N Tsukioka T Komatsu H Iwata T. Clinical Significance of Multiple Pulmonary Metastasectomy for Hepatocellular Carcinoma. World J Surg (2016) 40(2):380–7. 10.1007/s00268-015-3213-3 26306890

[21] Chen F Sato K Fujinaga T Sonobe M Shoji T Sakai H. Pulmonary Resection for Metastases From Hepatocellular Carcinoma. World J Surg (2008) 32(10):2213–7. 10.1007/s00268-008-9684-8 18668285

[22] Kawamura M Nakajima J Matsuguma H Horio H Miyoshi S Nakagawa K. Surgical Outcomes for Pulmonary Metastases From Hepatocellular Carcinoma. Eur J Cardiothorac Surg (2008) 34(1):196–9. 10.1016/j.ejcts.2008.03.056 18455409

